# Supplementary material for: Statistically derived geometrical landscapes capture principles of decision-making dynamics during cell fate transitions
Source: Cell Syst. 2022 Jan 19;13(1):12–28.e3. doi: 10.1016/j.cels.2021.08.013 (PMC8785827; doi:10.1016/j.cels.2021.08.013)
Supplement: Document S1. Figures S1–S5 and Table S1 [file mmc1.pdf]

Cell Systems, Volume *12*

Supplemental information

Statistically derived geometrical landscapes capture principles of decision-making dynamics during cell fate transitions

Meritxell Sáez, Robert Blassberg, Elena Camacho-Aguilar, Eric D. Siggia, David A. Rand, and James Briscoe

Supplemental Figures and Tables

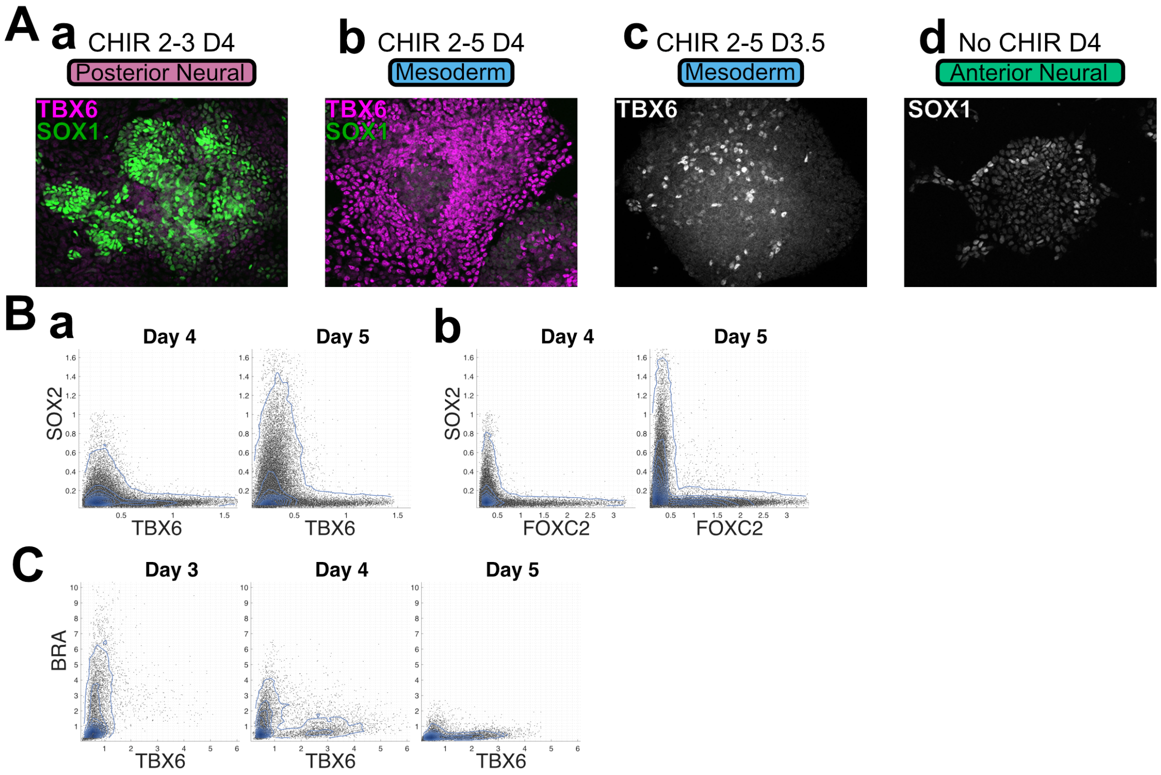

Figure S1: Spatiotemporal analysis of posterior neural and paraxial mesoderm differentiation

(A) Immunofluorescence assays indicate that CE progenitors at D4 adopt either SOX1 expressing posterior neural identity under the transient WNT signalling regime (Aa), or TBX6+ paraxial mesoderm identity under the sustained CHIR signalling regime (Ab). The expression of both TBX6 (Ac) and SOX1 (Ad) is initially not spatially organized. (B) Flow cytometry analysis of individual progenitors indicates that SOX2 levels are inversely correlated with both TBX6 (Ba) and FOXC2 (Bb) over the period of paraxial mesoderm differentiation. (C) Flow cytometry analysis indicates that the increase in the proportion of TBX6+ paraxial mesoderm progenitors over time is correlated with the loss of BRA+ paraxial mesoderm precursors.

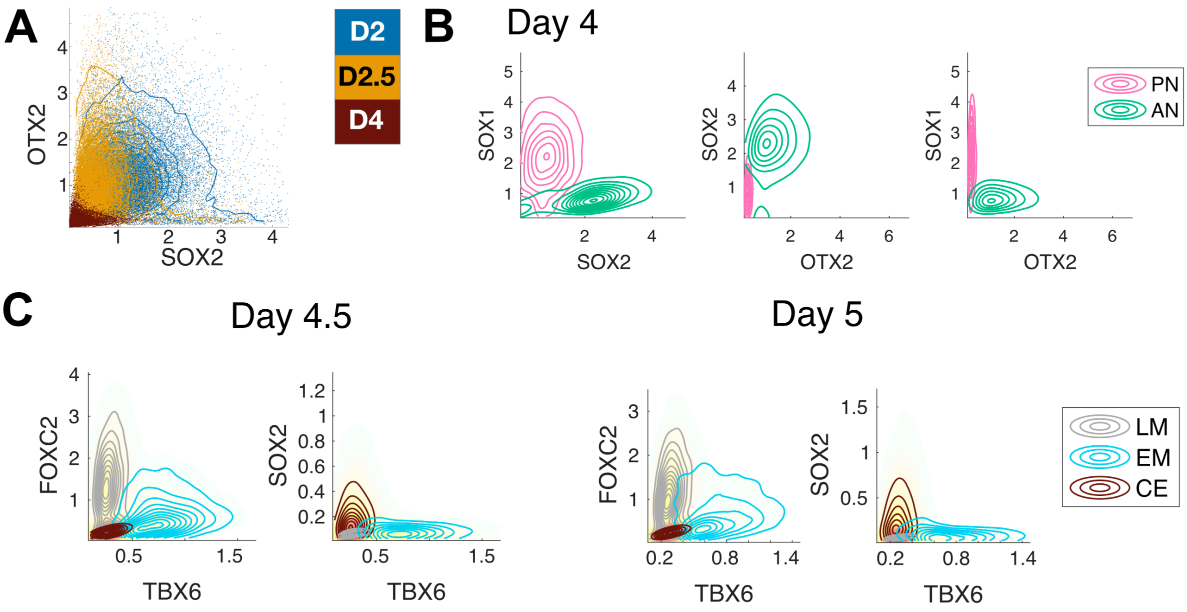

Figure S2: Distinct levels of SOX2 expression in cell types identified by clustering

(A) Flow cytometry analysis of individual progenitors exposed to CHIR from D2 indicates SOX2 expression is reduced to intermediate levels by D2.5, whereas OTX2 remains expressed. SOX2 is further downregulated by D4 under the sustained CHIR by which point OTX2 expression is also repressed.

(B) Clustering of the reference set of D4 samples with markers CDX2, OTX2, SOX1, SOX2. The clustering method identifies OTX2+ AN and OTX2 - PN clusters which express distinct levels of SOX1 and SOX2 with distributions that are almost identical to those obtained by the clusters defined with the minimal set of 5 markers used for model-fitting (Fig. 2B).

(C) Clustering of D4.5 and D5 samples exposed to continuous CHIR induction assayed with CDX2, FOXC2, SOX1, SOX2, TBX6. The clustering identifies a late mesodermal population expressing high levels of FOXC2 and very low levels of SOX2.

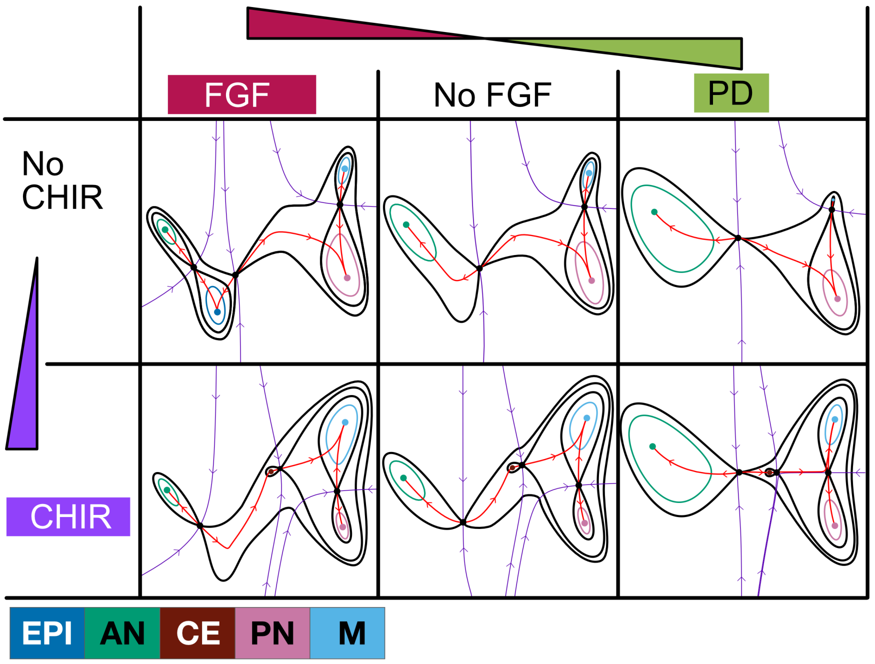

Figure S3: Landscape geometry and the effect of signalling after initial fitting

The landscapes produced by different combinations of signals are portrayed in the table. The parameters obtained by the initial fitting are used for the plot. The changes in the landscape that result from different signal combinations arise by bifurcations of attractors and flips in dynamical trajectories (unstable manifolds) of the parameterised landscape family. Note that the landscape corresponding to No CHIR+PD (top right) is an extrapolation of the fitting and not based directly on data, it therefore represents an untested prediction of the model (see Fig. 10). Colours correspond to cell identities as detailed. Average values from parameter distributions were used for the plot: No CHIR + FGF (-0.29, 1.17, -2.26, 0.66), CHIR + FGF (0.32, 0.41, -1.37, -0.52), CHIR + PD (-3, -8.44, -1.45, -0.0042) and corresponding linear combinations.

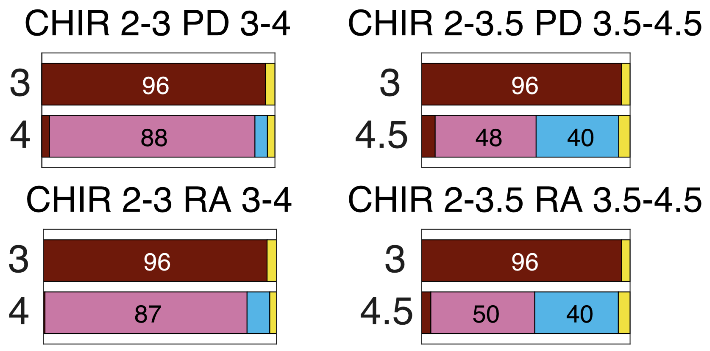

Figure S4: Proportions of cell types obtained in response to the indicated conditions

Comparison of RA and PD (FGF inhibition) treatments indicates that the cell type proportions are almost identical, suggesting they have equivalent effects on this system.

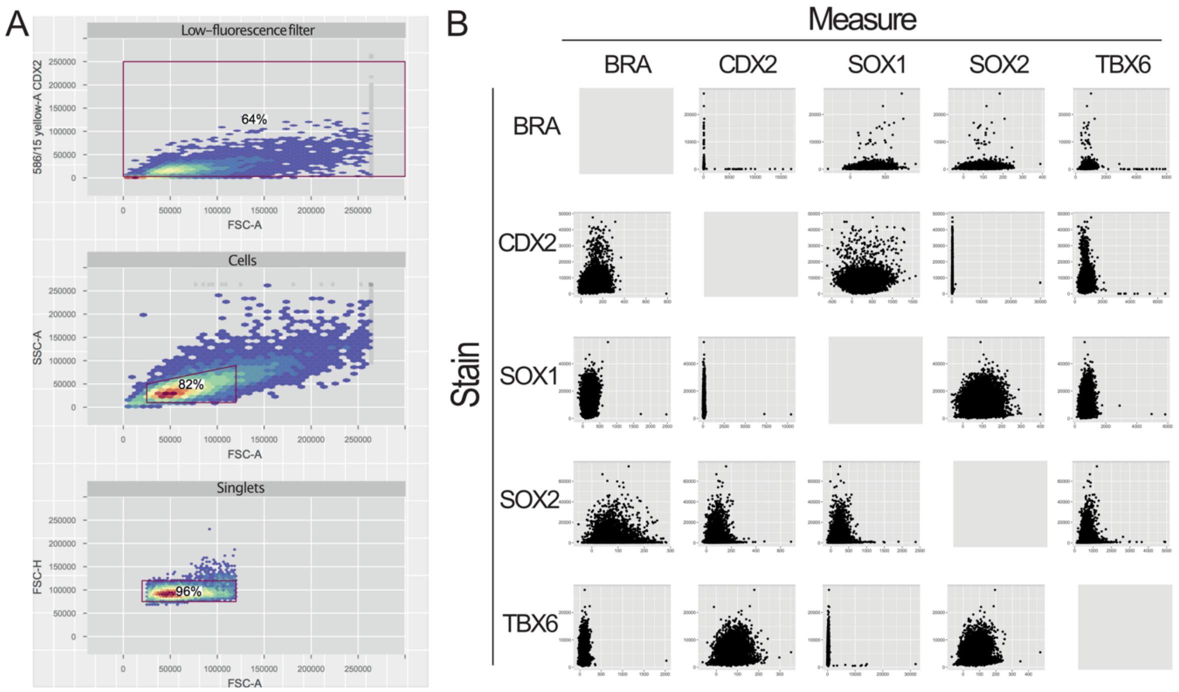

Figure S5: Flow Cytometry data preprocessing

(A) Illustration of the 'FlowCore' gating series applied to flow-cytometry data to identify single-cells for analysis. Removing events with extreme low values in the 586/15 channel improved subsequent clustering.

(B) Fluorescence measurements from samples stained with each of the 5 markers individually. Fluorescence was acquired in all five analysis channels and compensation was applied to account for the spectral overlap between fluorochromes. Appropriate compensation is demonstrated by the absence of spurious correlations between marker signals.

Name	Proteins	Conditions	Chiron time	FGF time	PD time	Measurement Days	Use
Dataset A	Bra, Cdx2, Sox1, Sox2, Tbx6	**No Chir**	--	0-3	--	2, 2.5, 3, 3.5 ,4, 4.5, 5	Training
Initial Exp. Series		Chir 2-2.5	2-2.5	0-3	--	2, 2.5, 3, 3.5 ,4, 4.5, 5	Validation
		**Chir 2-3**	2-3	0-3	--	2, 2.5, 3, 3.5 ,4, 4.5, 5	Training
		Chir 2-3.5	2-3.5	0-3	--	2, 2.5, 3, 3.5 ,4, 4.5, 5	Validation
		Chir 2-4	2-4	0-3	--	2, 2.5, 3, 3.5 ,4, 4.5, 5	Training
		**Chir 2-5**	2-5	0-3	--	2, 2.5, 3, 3.5 ,4, 4.5, 5	Training
		Ch 2-5 FGF 0-3	2-5	0-3	3-5	2, 2.5, 3, 3.5 ,4, 4.5, 5	Training
		Ch 2-5 FGF 0-3.5	2-5	0-3.5	3.5-5	2, 2.5, 3, 3.5 ,4, 4.5, 5	Validation
		Ch 2-5 FGF 0-4	2-5	0-4	4-5	2, 2.5, 3, 3.5 ,4, 4.5, 5	Training
		Ch 2-5 FGF 0-4.5	2-5	0-4.5	4.5-5	2, 2.5, 3, 3.5 ,4, 4.5, 5	Validation
		Ch 2-5 FGF 0-5	2-5	0-5	--	2, 2.5, 3, 3.5 ,4, 4.5, 5	Training
Dataset B1	Bra, Cdx2, Sox1, Sox2, Tbx6	**No Chir**	--	0-3	--	2, 2.5, 3, 3.5 ,4, 4.5, 5	Training 2
Test Exp. Series		Chir 2-2.5	2-2.5	0-3	--	2, 2.5, 3, 3.5 ,4, 4.5, 5	Training 2
		**Chir 2-3**	2-3	0-3	--	2, 2.5, 3, 3.5 ,4, 4.5, 5	Training 2
		**Chir 2-5**	2-5	0-3	--	2, 2.5, 3, 3.5 ,4, 4.5, 5	Training 2
		Ch 1 Pulse		0-3	--	2, 2.5, 3, 3.5 ,4, 4.5, 5	Training 2
		Ch 2 Pulses		0-3	--	2, 2.5, 3, 3.5 ,4, 4.5, 5	Training 2
		Chir 2-3 0.1	2-3 (1) 3-5(0.1)	0-3	--	2, 2.5, 3, 3.5 ,4, 4.5, 5	DR Curve
		Chir 2-3 0.3	2-3 (1) 3-5(0.3)	0-3	--	2, 2.5, 3, 3.5 ,4, 4.5, 5	DR Curve
		Chir 2-3 0.5	2-3 (1) 3-5(0.5)	0-3	--	2, 2.5, 3, 3.5 ,4, 4.5, 5	DR Curve
Dataset B2	Cdx2, Otx2, Sox1, Sox2	**No Chir**	--	0-3	--	2, 2.5, 3, 3.5 ,4, 4.5, 5	Training 2
Test Exp. Series		Chir 2-2.5	2-2.5	0-3	--	2, 2.5, 3, 3.5 ,4, 4.5, 5	Training 2
		**Chir 2-3**	2-3	0-3	--	2, 2.5, 3, 3.5 ,4, 4.5, 5	Training 2
		**Chir 2-5**	2-5	0-3	--	2, 2.5, 3, 3.5 ,4, 4.5, 5	Training 2
		Ch 1 Pulse		0-3	--	2, 2.5, 3, 3.5 ,4, 4.5, 5	Training 2
		Ch 2 Pulses		0-3	--	2, 2.5, 3, 3.5 ,4, 4.5, 5	Training 2
		Chir 2-3 0.1	2-3 (1) 3-5(0.1)	0-3	--	2, 2.5, 3, 3.5 ,4, 4.5, 5	DR Curve
		Chir 2-3 0.3	2-3 (1) 3-5(0.3)	0-3	--	2, 2.5, 3, 3.5 ,4, 4.5, 5	DR Curve
		Chir 2-3 0.5	2-3 (1) 3-5(0.5)	0-3	--	2, 2.5, 3, 3.5 ,4, 4.5, 5	DR Curve
Dataset B3	Cdx2, FoxC2, Sox1, Sox2, Tbx6	Chir 2-5	2-5	0-3	--	2.5, 3, 3.5 ,4, 4.5, 5	Validation
Dataset C	Otx2, Cdx2, Sox1, Sox2	**No Chir**	--	0-3	--	3, 4, 5	
Prediction 1 Exp. Series		**Chir 2-3**	2-3	0-3	--	3, 4, 5	
		Chir 4-5	4-6	0-3	--	3, 4, 5	Prediction
Dataset D	Bra, Cdx2, Sox1, Sox2, Tbx6	**Chir 2-3**	2-3	0-3	--	3, 4, 5	
Prediction 2 Exp. Series		Chir 2-3.5	2-3.5	0-3	--	3, 4.5	
		Chir 2-4	2-4	0-3	--	3, 4, 5	
		**Chir 2-5**	2-5	0-3	--	3, 4, 5	
		Chir 2-3 PD 3-4	2-3	0-3	3-4	3, 4	Prediction
		Chir 2-3 RA 3-4	2-3	0-3	RA 3-4	3, 4	Prediction
		Chir 2-3.5 PD 3.5-4.5	2-3.5	0-3	3.5-4.5	3, 4.5	Prediction
		Chir 2-3.5 RA 3.5-4.5	2-3.5	0-3	RA 3.5-4.5	3, 4.5	Prediction
		Chir 2-4 RA 4-5	2-4	0-3	RA 4-5	3, 4, 5	Prediction

Table S1 Datasets: All datasets used in this work are described with the corresponding information on proteins measured by flow cytometry, detailed signalling protocol for each condition, measurement time points and specific use in this work.
